# First field efficacy trial of the ChAd63 MVA ME-TRAP vectored malaria vaccine candidate in 5-17 months old infants and children

**DOI:** 10.1371/journal.pone.0208328

**Published:** 2018-12-12

**Authors:** Alfred B. Tiono, Issa Nébié, Nicholas Anagnostou, Aboubacar S. Coulibaly, Georgina Bowyer, Erika Lam, Edith C. Bougouma, Alphonse Ouedraogo, Jean Baptist B. Yaro, Aïssata Barry, Rachel Roberts, Tommy Rampling, Carly Bliss, Susanne Hodgson, Alison Lawrie, Amidou Ouedraogo, Egeruan Babatunde Imoukhuede, Katie J. Ewer, Nicola K. Viebig, Amidou Diarra, Odile Leroy, Philip Bejon, Adrian V. S. Hill, Sodiomon B. Sirima

**Affiliations:** 1 Centre National de Recherche et de Formation sur le Paludisme, Ouagadougou, Burkina Faso; 2 The Jenner Institute, Nuffield Department of Clinical Medicine, University of Oxford, Oxford, United Kingdom; 3 European Vaccine Initiative, Universitäts Klinikum Heidelberg, Heidelberg, Germany; 4 Kenya Medical Research Institute-Wellcome Trust Research Programme, Kilifi, Kenya; 5 Groupe de Recherche Action en Santé (GRAS), Ouagadougou, Burkina Faso; Public Health England, UNITED KINGDOM

## Abstract

**Background:**

Heterologous prime boost immunization with chimpanzee adenovirus 63 (ChAd63) and Modified Vaccinia Virus Ankara (MVA) vectored vaccines is a strategy previously shown to provide substantial protective efficacy against *P*. *falciparum* infection in United Kingdom adult Phase IIa sporozoite challenge studies (approximately 20–25% sterile protection with similar numbers showing clear delay in time to patency), and greater point efficacy in a trial in Kenyan adults.

**Methodology:**

We conducted the first Phase IIb clinical trial assessing the safety, immunogenicity and efficacy of ChAd63 MVA ME-TRAP in 700 healthy malaria exposed children aged 5–17 months in a highly endemic malaria transmission area of Burkina Faso.

**Results:**

ChAd63 MVA ME-TRAP was shown to be safe and immunogenic but induced only moderate T cell responses (median 326 SFU/10^6^ PBMC (95% CI 290–387)) many fold lower than in previous trials. No significant efficacy was observed against clinical malaria during the follow up period, with efficacy against the primary endpoint estimate by proportional analysis being 13.8% (95%CI -42.4 to 47.9) at sixth month post MVA ME-TRAP and 3.1% (95%CI -15.0 to 18.3; p = 0.72) by Cox regression.

**Conclusions:**

This study has confirmed ChAd63 MVA ME-TRAP is a safe and immunogenic vaccine regimen in children and infants with prior exposure to malaria. But no significant protective efficacy was observed in this very highly malaria-endemic setting.

**Trial registration:**

ClinicalTrials.gov NCT01635647.

Pactr.org PACTR201208000404131.

## Introduction

Malaria is the preeminent tropical infectious disease globally, with a devastating effect on human health and society. In 2016, an estimated 216 million cases of malaria occurred worldwide (95% CI 196–263 millions) with an estimated 445 000 deaths [1]. Progress in reducing transmission in Sub-Saharan Africa has stalled recently [2]. The enormous economic and social consequences of malaria have been well documented. The development of a vaccine against malaria is a high priority and of significant importance in the context of coordinated efforts to reduce the burden of malaria. The development of an effective vaccine is likely necessary for the global eradication of malaria [3] and strategic goals for vaccine developers are described in the malaria vaccine technology roadmap [4].

The most advanced malaria vaccine, which received a positive opinion under article 58 from the European Medicine Agency (EMA), RTS,S/AS01, targets antibodies against the circumsporozoite protein (CS), which is expressed by the sporozoite at the pre-erythrocytic stage. A Phase III trial with RTS,S/AS01E showed modest efficacy of 28·3% (95% CI 23·3–32·9%) against clinical malaria in children 5–17 months old who received three doses and of 36·3% (31·8–40·5%) in those given a fourth dose, during 48 months of follow-up. A much lower efficacy was observed in young infants [5,6]. The need for four doses with three outside current EPI time points and the unexplained imbalance of female mortality between vaccine recipients and controls (nearly doubled female mortality in vaccinees) has led to a need identified by WHO for very large deployment trials to assess potential suitability for WHO pre-qualification of this vaccine. The malaria vaccine “implementation” programme should start in late 2018 and will last many years and will evaluate several aspects such as the operational feasibility, the Impact in mortality (overall and by gender) and the safety (Adverse Events Following Immunization with emphasis in meningitis and cerebral malaria). [7].

The only other subunit malaria vaccination approach that has demonstrated repeatable partial efficacy in humans involves the use of virally vectored vaccines containing a recombinant genetic insert, encoding the antigen against which the immune response is directed [8–10]. The malaria vaccine candidate ChAd63 ME-TRAP and MVA ME-TRAP consist of non-replicating viral vectors (ChAd63 and MVA) expressing the insert, ME-TRAP. Heterologous prime-boost vaccination with ChAd63 ME-TRAP prime, followed eight weeks later by MVA ME-TRAP boost, has shown partial sterile efficacy of 20–25% against *P*. *falciparum* infection in UK adult Phase IIa sporozoite challenge studies, and 67% efficacy against PCR-determined malaria infection in a trial in Kenyan adults [11]. T cell responses to TRAP peptides were associated with protection in these Kenyan adults consistent with a CD8^+^ T cell correlate identified in UK adult vaccines [8]. In a similar trial in Dakar Senegal, [9] lower T cell immunogenicity was observed and non-significant efficacy, although a protocol-specified combined meta-analysis of the similar Kenyan and Senegalese trials showed an overall efficacy of 50%. Age de-escalation trials involving Gambian and Burkinabe children and infants have, importantly, shown a higher level of T cell response than in UK adults in young Gambian infants administered these vaccine with and without concomitant administration of other infant vaccines [12–14].

Based on these promising findings, we undertook a safety, immunogenicity and efficacy trial of the two dose ChAd63 MVA /ME-TRAP malaria vaccination regime in malaria exposed older infants and young children aged between 5 and 17 months in an area of hyperendemic, seasonal and stable malaria transmission in South-Western Burkina Faso.

## Methods

### The trial site

The study was conducted in the Banfora trial site, which is located about 400 km from Ouagadougou, the capital city of Burkina Faso. The “Unité de Recherche Clinique de Banfora” (URC-B) research unit is located within the complex of the regional hospital. From recent surveys, the bed net coverage was 80%. There was no implementation of indoor residual spraying or intermittent preventive treatment (IPT) in infants or children in the area. To date in this area there is no evidence of decline in malaria incidence that has been recently reported from other parts of sub-Saharan Africa. In the study area, malaria is very highly endemic. Transmission occurs throughout the year, with a peak during the rainy season (from June to October). *P*. *falciparum* is responsible for more than 90% of all clinical malaria cases [15,16]. The major vectors are *Anopheles gambiae*, *An*. *arabiensis and An*. *funestus*. Entomological inoculation rates (EIR) vary from 55 to 400 infected bites/person/year (our unpublished data).

### Trial participants

The trial enrolled infants and children aged from 5 to 17 months at the time of first vaccination and whose parents or legally accepted guardians / representatives were permanently resident in the study area. They were drawn from six community clinic’s catchment areas of Banfora health district. There was no selection on the basis of pre-existing neutralizing antibodies (NAb) against ChAd63 vector prior to enrolment: antibody levels to this vector are low in Burkina Faso, as described elsewhere [17] and previous work has found no evidence of any impact of such low level response on vaccine immunogenicity. Volunteers were considered eligible if their parents or legally accepted guardians / representatives had provided written informed consent and were likely to remain resident in the study area for the trial duration. Exclusion criteria included any evidence of acute or chronic illness or hematological, hepatic or renal pathology. Specific exclusion criteria included prior receipt of an investigational malaria vaccine, recent or planned used of an investigational drug, vaccine, immunoglobulin or any blood product, confirmed or suspected immunodeficiency history, surgical splenectomy, concurrent participation in another clinical trial or participation within 3 months of this study. The full list of inclusion and exclusion criteria is given in the study protocol (S1 Protocol).

### Study vaccines

#### The malaria vaccine candidates ChAd63 ME-TRAP and MVA ME-TRAP

The recombinant vectors and their generation have been described previously [8]. The antigen ME-TRAP is a fusion protein of a multi-epitope string (ME), followed by the pre-erythrocytic thrombospondin related adhesion protein (TRAP) from *P*. *falciparum* strain T9/96 [8]. “ME” is a string of 20 epitopes, mainly CD8 T cell epitopes, from *P*. *falciparum* pre-erythrocytic antigens fused to the TRAP protein. These epitopes are from six *P*. *falciparum* target antigens and are included with the aim to broaden the immune responses in vaccinated volunteers.

The ChAd63 ME-TRAP and MVA ME-TRAP were both manufactured under Good Manufacturing Practice conditions. The ChAd63 ME-TRAP was manufactured by the Clinical Biomanufacturing Facility (CBF), Churchill Hospital, Oxford. ChAd63 ME-TRAP is supplied as a sterile 0.5–1.0 ml liquid in 2.0ml glass vials. The dose of ChAd63 ME-TRAP used in this study was 5 x 10^10^ vp. The MVA ME-TRAP was manufactured by IDT Biologika GmbH (IDT), Germany. MVA ME-TRAP was supplied as a sterile 0.55ml liquid in 2.0 mL transparent glass injection vials. The dose of MVA ME-TRAP used was 1 × 10^8^ pfu.

#### The rabies control vaccine Imovax

The Imovax Rabies Vaccine produced by Sanofi Pasteur SA is a sterile, stable, freeze-dried suspension of rabies virus prepared from strain PM-1503-3M obtained from the Wistar Institute, Philadelphia, PA. The vaccine is supplied as a single dose vial to be administered intramuscularly. The rabies vaccine was chosen as the most potentially beneficial active control vaccine for the study participants.

### Study procedures

This study was a Phase IIb, double blind, randomized controlled trial. The primary objective was to assess the protective efficacy against clinical malaria of ChAd63 ME-TRAP / MVA ME-TRAP prime-boost immunization, in 5–17 months old infants and children living in a malaria-endemic area, for 6 months after the last vaccination.

The trial, participants were randomized 1:1 to receive the candidate malaria vaccine (ChAd63 ME-TRAP and MVA ME-TRAP) or control vaccination with two doses of a rabies vaccine (Imovax Sanofi Pasteur).

The prime-boost regimen was given with an eight-week interval between doses. ChAd63 ME-TRAP was followed eight weeks later by MVA ME-TRAP. Both vaccinations were by intramuscular route at doses of 5 x 10^10^vp and 1 x 10^8^pfu, respectively, both to the anterolateral thigh.

ChAd63 ME-TRAP and MVA ME-TRAP are non-replicating genetically modified organisms. Although previous work has failed to identify any evidence of dissemination of these replication-incompetent vectors, to minimize any possibility of dissemination of the vectors into the environment, the inoculation site for all vaccinations (including the rabies vaccine to maintain the blinding) were covered with a dressing after immunization to absorb any vector that may leak out through the needle track. The dressing was removed from the injection site after 30 minutes. Each volunteer was monitored for one hour (or longer if necessary) after each vaccination.

Monitoring of solicited adverse events was performed for seven days after each vaccination. Unsolicited adverse events were recorded until one month post each vaccination. Serious adverse events and malaria episodes were monitored throughout the study duration.

Trained field workers (qualified nurses) under the supervision of the study clinicians visited daily each enrolled child from days 1 to 3 after each vaccination. If necessary, the child continued to be seen by the field worker on subsequent days for follow-up of any adverse event. If any severe (grade 3) event was observed, the volunteer was brought to the vaccination center for examination by a study physician.

When a study participant was unwell at any time during the study, the parents were advised to report to the trial site clinic or the nearest health facility for follow up where the study medical staff were available 24 hours a day to identify study participants through the personal identification card and to ensure standardized documentation and appropriate medical management. A duplicate blood film was obtained if the volunteer had symptoms or signs compatible with malaria (axillary temperature ≥ 37.5°C, history of fever within the last 24 hours, loss of appetite, malaise, vomiting and diarrhea, etc.). A malaria rapid diagnosis test was performed for prompt management of the child while the duplicate blood film was read later to quantify the *P*. *falciparum* parasite density. The study participants with clinical conditions requiring hospitalization were referred to the Pediatric ward of the regional hospital of Banfora for admission and further clinical and laboratory investigations. The clinical management was performed in accordance with local standard of care and national guidelines.

### Laboratory evaluations

#### Ex-vivo interferon gamma (IFNγ) enzyme-linked immunosorbent spot (ELISPOT) analysis

The kinetics and magnitude of the T cell response to ME-TRAP were assessed over time by ex-vivo IFNγ ELISPOT assays performed on blood samples taken at days 0, 63, and 243. Ex-vivo IFNγ ELISPOT assays were performed with an 18–20 hour stimulation of peripheral blood mononuclear cells (PBMC) with peptides pools containing up to 10 peptides per pool, including peptides representing the T9/96 and 3D7 strains of *P*. *falciparum* TRAP. Fresh PBMC were used in all ELISPOT assays using a previously described protocol [18], except that 50 μL/well ME-TRAP peptide pools (final concentration of each peptide 10 μg/mL) were added to duplicate wells, 50 μL/well of medium only and DMSO control were added to negative un-stimulated wells, and 50 μL/ well Staphylococcal enterotoxin B (SEB) (final concentration 0.02 μg/mL) plus phytohemagglutinin (PHA) (final concentration 10 μg/mL) was added to positive control wells. Spots were counted using an ELISPOT counter (Autoimmun Diagnostika (AID), Germany). Results are expressed as the mean of the duplicate IFNγ spot-forming cells (SFC) per million PBMC. Background responses in un-stimulated control wells were subtracted from those measured in peptide-stimulated wells. Responses are shown as the summed response to all the ME-TRAP (T9/96) peptide pools.

To establish parasite presence and density of *P falciparum* asexual stages, Giemsa-stained blood slides were read following standard quality-controlled procedures.

The presence of parasites on capillary blood samples was assessed by 100 x bright field microscopic examination, assuming 8000 leukocytes/μl of blood. The count was made by species (*P*. *falciparum*, *P*. *malariae*, or *P*. *ovale*), and counts for *P*. *falciparum* were made for both sexual and asexual parasites. The parasite presence and density were determined independently by two readers for the same slide; if readings were judged to be discordant, a third independent read was organized. The parasite density (parasites/μL) was calculated as the geometric mean of the two positive readings (two geometrically closest readings in the case of three positive reads).

#### TRAP-specific total IgG ELISA

Standardized ELISAs for TRAP-specific antibodies were conducted as previously described [10] using recombinant TRAP protein as antigen. Briefly, a reference standard of pooled anti-TRAP antibody positive serum was serially diluted to produce a standard curve, which was included on all plates. The standard sample was assigned a value in arbitrary ELISA units (EUs). The standard curve was then used to convert absorbance values of individual test sera (diluted to fall within the linear range of the curve) into EUs. A “seropositive cut-off” value was calculated using the mean plus three standard deviations of the EU values of 42 serum samples from unvaccinated UK volunteers. For the total IgG standardized ELISA, this cut-off value was 88 EUs.

### Statistical analysis

An efficacy of vaccination of 30% was that considered suitable for this vaccine strategy to be potentially included in a future multi-component high efficacy vaccine. With a total sample of 700 participants, the expected power to detect 30% vaccine efficacy, (i.e., a hazard ratio of 0.7, with the proportion of malaria in malaria vaccine candidate recipients by Day 243 equaling 38%) was 88%.

The proportion of children remaining free of any episode of *P*. *falciparum* malaria was calculated by the Kaplan-Meier estimate to determine the proportions free by vaccination group (i.e. proportion among vaccinees (Pv) and proportion among controls (Pc)). Vaccine efficacy was determined as 100 x (1- Pv/Pc)%. Confidence intervals were calculated on a log scale.

For analysis of first or only episodes of *P*. *falciparum* malaria, the incidence of episodes (episodes/person years at risk) for each group were calculated. Kaplan-Meier curves for both groups are shown. The distribution of the survival time was compared with the Wilcoxon test (if efficacy appears to vary with time) or the Log-rank test (if it does not). Vaccine efficacy was assessed using Cox regression models for the first episode. Vaccine efficacy is defined as 1- R where R is the hazard ratio of the malaria vaccine group versus the control group (with 95% CI).

The Kaplan-Meier plots were examined for evidence contradicting the assumption of proportionality of hazards. A test based on the Schoenfeld residuals was performed for proportionality of the hazard.

Secondary analyses have examined multiple episodes, using the robust clustering method by individual, with examination of proportionality of hazards and calculation of efficacy as described above.

Subgroup analysis was conducted by age strata (i.e. 5–12 months and 12–17 months of age). Variation in efficacy by age was tested by an interaction between age and vaccine efficacy, using these categories and using age as a continuous variable.

Variation in efficacy according to immunogenicity was assessed, using ELISPOT outcomes, with log transformation to ensure normal distributions, and analyzing only the vaccinees to test for variation in risk using ELISPOT data as a continuous explanatory variable.

Analyses were done with Stata version STATA software (Version 15.0, College Station, TX: StataCorp; 2017).

### Ethical and regulatory approvals

The clinical trial protocol and associated documents were reviewed and approved by CNRFP institutional bioethics committee (approval reference N° 2012/04/MS/SG/CNRFP/CIB), the Ministry of Health Ethical Committee for Biomedical Research (approval reference N° 2012-6-37) and Oxford Tropical Research Ethics Committee (OXTREC) (approval reference 41–12). Regulatory approval was given in Burkina Faso by the National Regulatory authority (Comité Technique pour les Essais Cliniques, CTEC). All study participants’ parents or legally acceptable representatives gave documented informed consent before any study procedures were performed. The trial was conducted according to the principles of the declaration of Helsinki and International Conference on Harmonization (ICH) Good Clinical Practice (GCP) guidelines. An independent data safety monitoring board (DSMB) and local safety monitors provided safety oversight and GCP compliance was independently monitored by an external organization (AppleDown Clinical Research Ltd, Great Misseden, UK).

## Results

### Recruitment and vaccination

**Fig 1:**
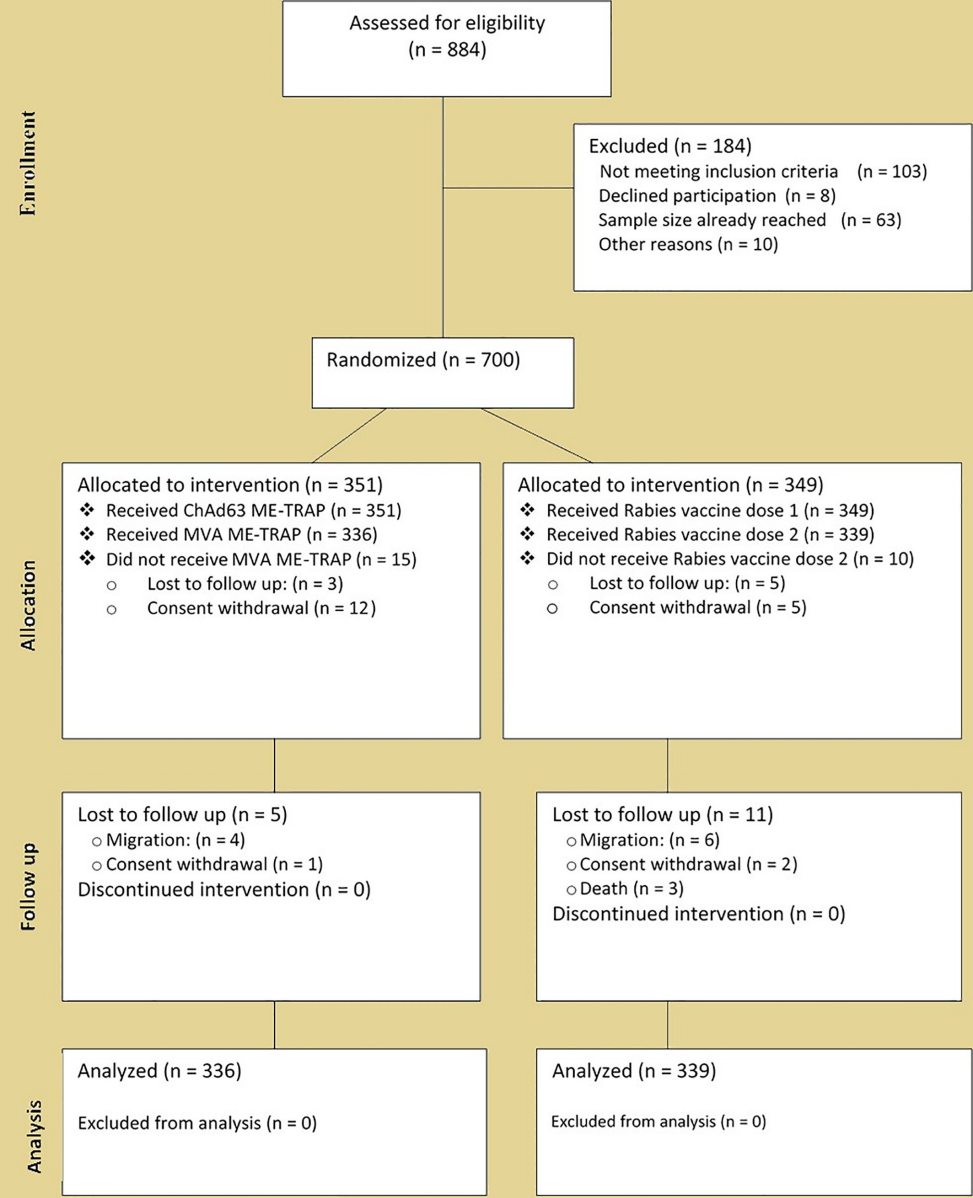
Trial profile.

Recruitment took place between 18^th^ March 2013 and 14 June 2013. 884 children were screened to enroll 700, from whom 351 received ME-TRAP vaccines and 349 received rabies vaccine. The mean age of study participants was 10.6 months (range 5 to 17.9 months). 334 children received 2 doses of ME-TRAP vaccine and have follow up data, 336 received 2 doses of rabies vaccine and have follow up data and are included in the according to protocol (ATP) analysis. 42 children did not complete the follow up among them 20 consent withdrawals (none related to adverse event), 10 migrations/lost to follow up, and 12 for other reasons such as absence at the time of end of study visit and deaths (4 participants). The study profile is summarized in Fig 1.

### Vaccine safety and reactogenicity

**Fig 2:**
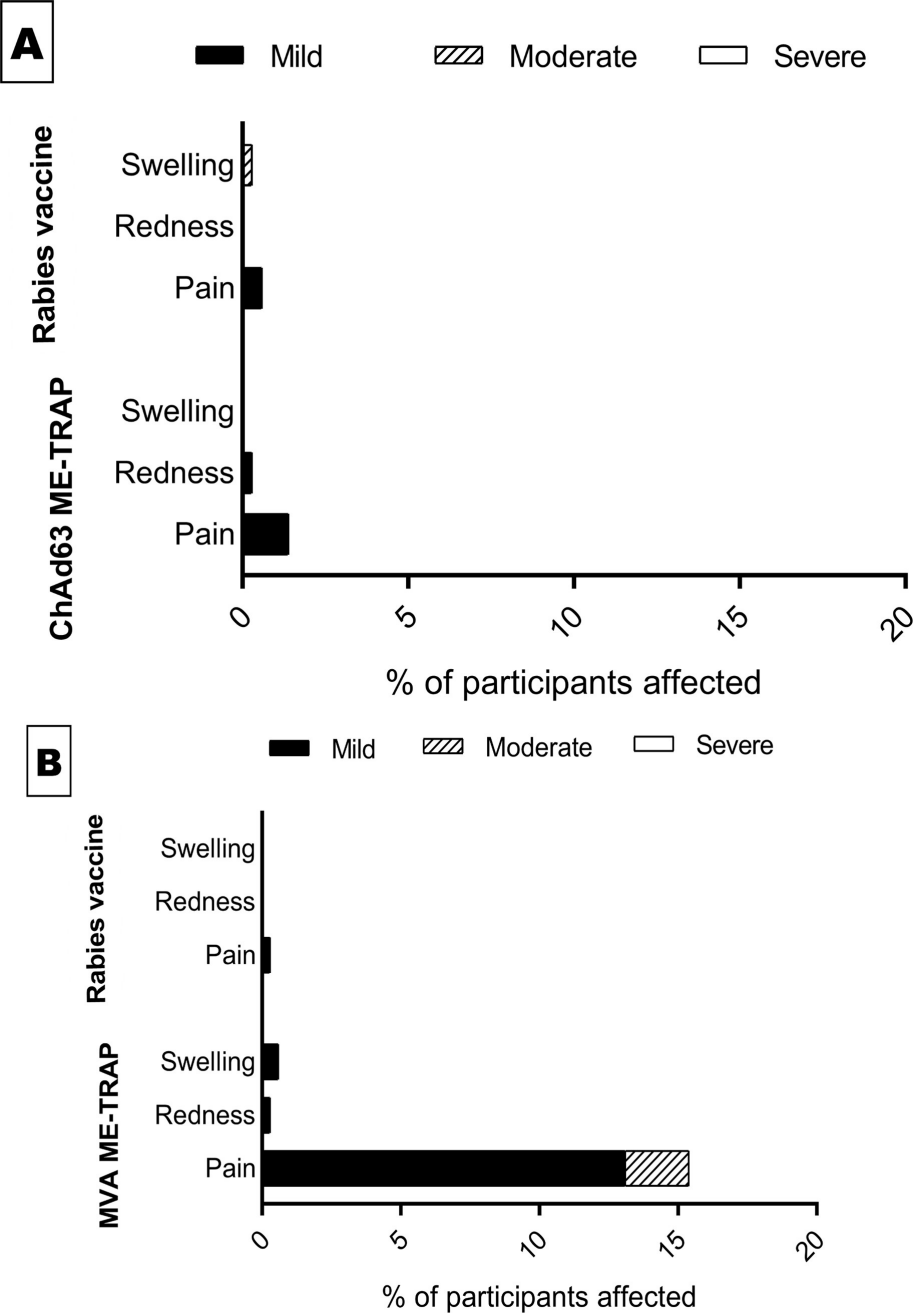
Incidence of local solicited adverse events in trial participants. **(A)** Local AEs post ChAd63 ME-TRAP vs. Rabies. **(B)** Local AEs post MVA ME-TRAP vs. Rabies.

The local and systemic reactogenicity profiles are summarized in (Fig 2A & 2B). Except for more local pain in the malaria vaccine group for solicited local symptoms (15.4% in the malaria vaccine vs. 0.3% of rabies vaccine group), no clear difference in reactogenicity was noted between participants receiving rabies and ME-TRAP vaccines. The highest frequency of local pain was recorded after the MVA ME-TRAP vaccination. Most of the local solicited symptoms recorded were mild to moderate and resolved within one week.

**Fig 3:**
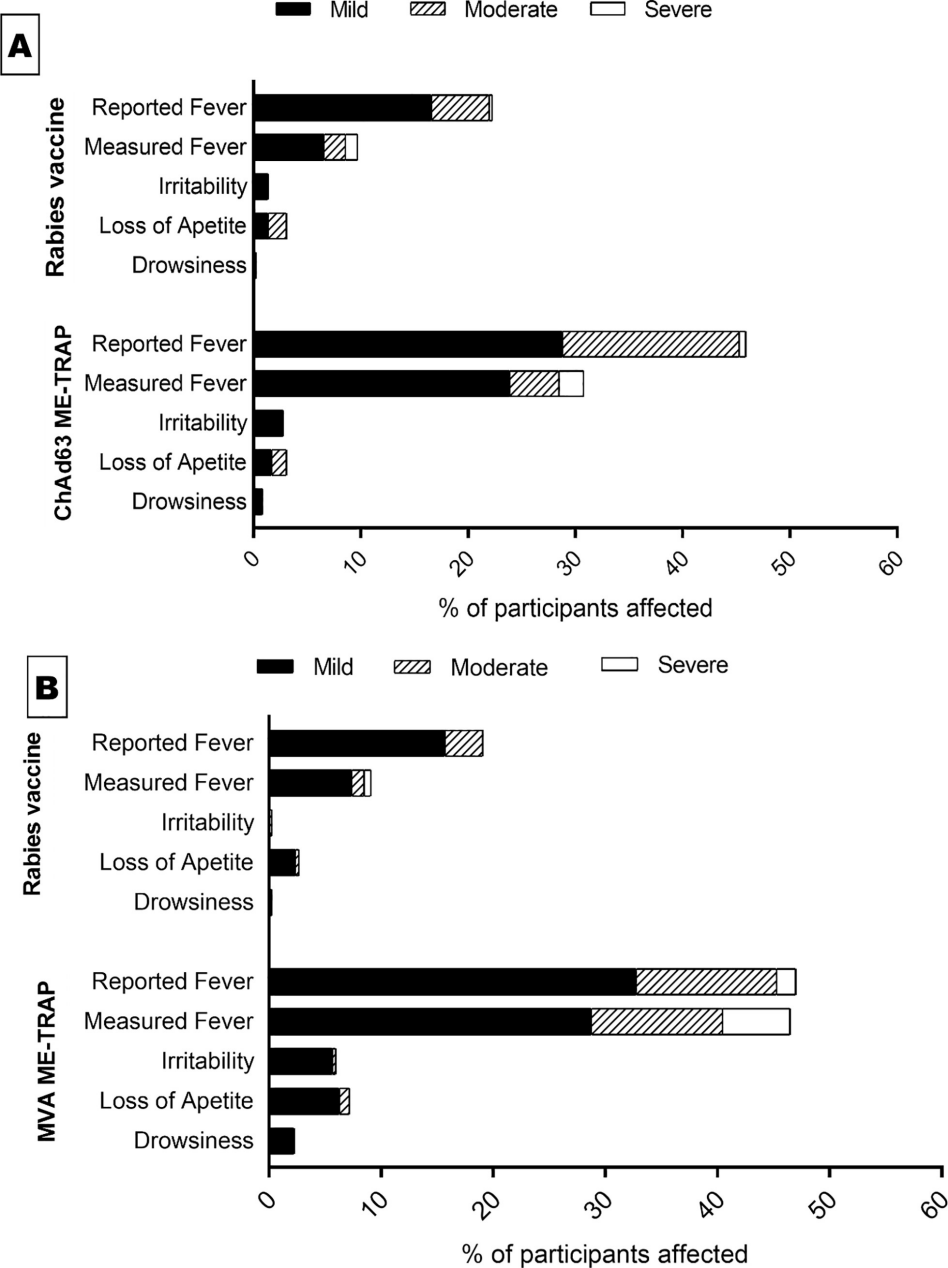
Incidence of solicited systemic adverse events in trial participants. **(A)** Solicited systemic AEs post ChAd63 ME-TRAP vs. Rabies. **(B)** Solicited systemic AEs post MVA ME-TRAP vs. Rabies.

Solicited systemic symptoms were loss of appetite, irritability, drowsiness and fever (both measured and reported) (Fig 3A & 3B). ME-TRAP was more reactogenic than rabies vaccine though still well tolerated with most AEs being mild in intensity. Measured and reported fever followed similar trends with most of reported cases being mild to moderate in intensity and more prevalent in ME-TRAP vaccinees compared to the rabies vaccine group (measured fever occurred in 46.5% of malaria vaccine recipients versus 9.1% of rabies vaccine control recipients after the second dose while reported fever was recorded in 45.9% of malaria vaccine recipients versus 22.3% of rabies vaccine control recipients). Most of the solicited systemic symptoms recorded were also mild to moderate and resolved within one week.

On final analysis the most common Serious Adverse Events (SAEs) were pneumonia (12 cases in ME-TRAP vaccinees and 10 in rabies vaccine group), non-severe malaria (8 episodes in ME-TRAP group against 9 in rabies vaccine group), gastroenteritis (4 in ME-TRAP group and 3 in rabies vaccine group) and malnutrition (6 in ME-TRAP group and 2 in rabies vaccine group). In total 2 cases of sepsis of unknown causes were recorded during the trial; all in the rabies vaccine group.

There were 4 deaths recorded, one in the ME-TRAP vaccine recipients and three in the rabies vaccine recipients. The death in the ME-TRAP vaccinee was a case of sudden death that occurred 80 days after the MVA ME-TRAP vaccination at home while the male study participant was under self-medication for illness with traditional remedies. The three deaths in the rabies recipients were cases of severe malaria, sepsis in a context of acute moderate malnutrition, and cranial trauma. None of the deaths were temporally associated with the study vaccination. All the other serious adverse events recorded were not unexpected events given the study population and were not closely related to vaccination in timing. None were judged likely to be linked to vaccination by the investigators and the DSMB.

Hematological values outside the normal range were infrequent. There was no statistically significant difference in the median values for any parameter between the two groups post immunization.

Biochemistry values outside the normal range were also infrequent. One-month post MVA ME-TRAP vaccination, the median values for creatinine were statistically higher in the malaria vaccine recipients than in control vaccine recipients (31.2 vs 30.2 μmol/l, p = 0.017). The same trends towards higher values were observed 6 months later for creatinine (34.5 vs 32.9 μmol/l, p = 0.007) and bilirubin (9.0 vs. 8.0 μmol/l, p = 0.017). In total 21 comparisons of biochemical and hematological parameters were made (S1 Table).

### T-cell immunogenicity to ChAd63 MVA ME-TRAP

**Fig 4:**
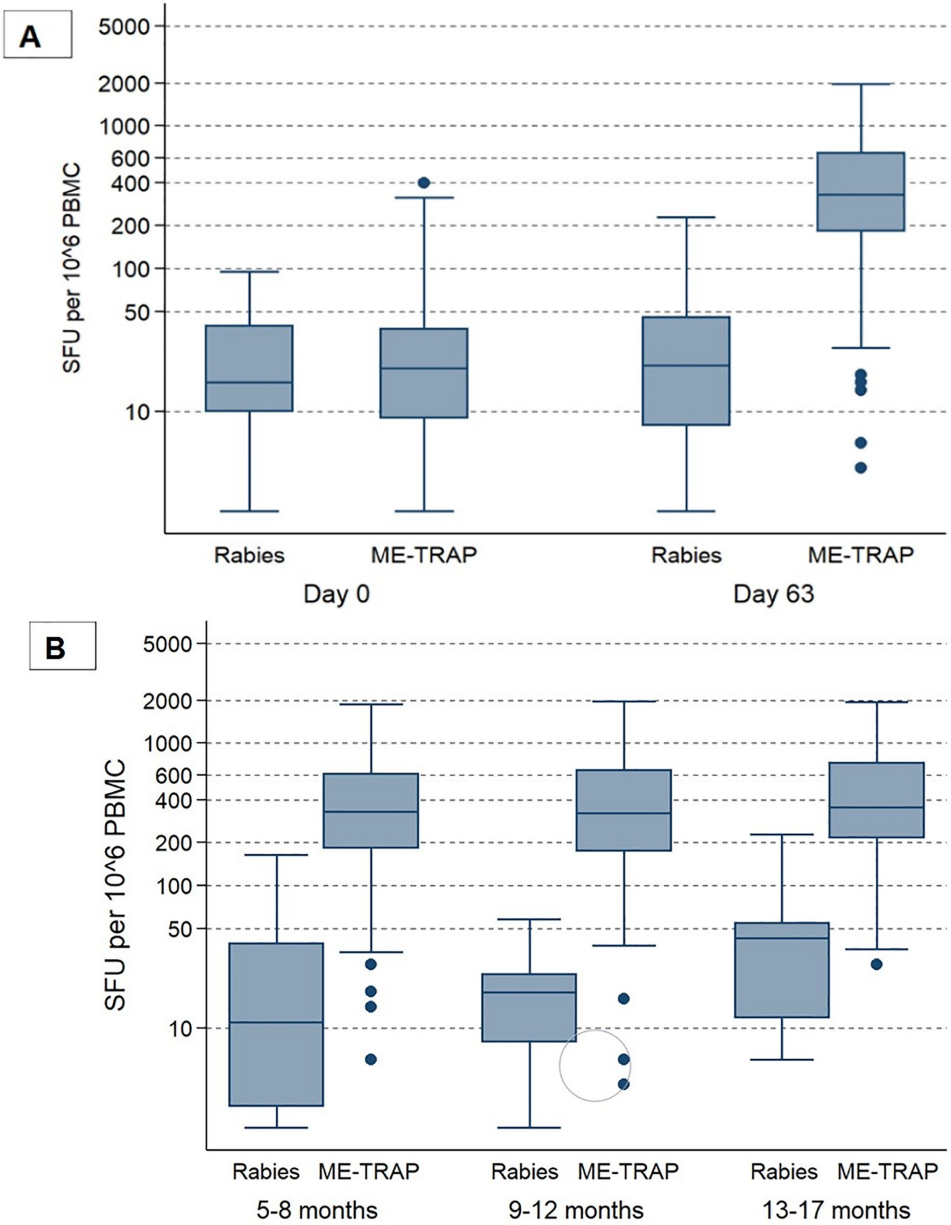
IFN-γ ELISPOT responses to ChAd63 MVA ME-TRAP. A total of 169 children were part of a nested cohort randomly selected by the study statistician for the IFN-γ ELISPOT responses evaluation **(A)** represents the responses at baseline and 7 days post MVA ME-TRAP and **(B)** the responses at day 63 according to age categories.

Heterologous prime boost vaccination with ChAd63 MVA ME-TRAP increased frequencies of antigen-specific IFNγ secreting T cells measured by ex-vivo IFNγ ELISPOT. Among ME-TRAP vaccinees, *ex vivo* T cell responses rose from a median of 16 SFC /10^6^ PBMC before vaccination (95% CI 14–18) to 326 SFC/10^6^ PBMC (95% CI 290–387) after the MVA vaccination (i.e. at day 63, Fig 4A). The median IFNγ ELISPOT responses at Day 63 by age group is presented in Fig 4B and there was no significant difference between age-groups after MVA ME-TRAP vaccination.

### Humoral response to ChAd63 MVA ME-TRAP

**Fig 5:**
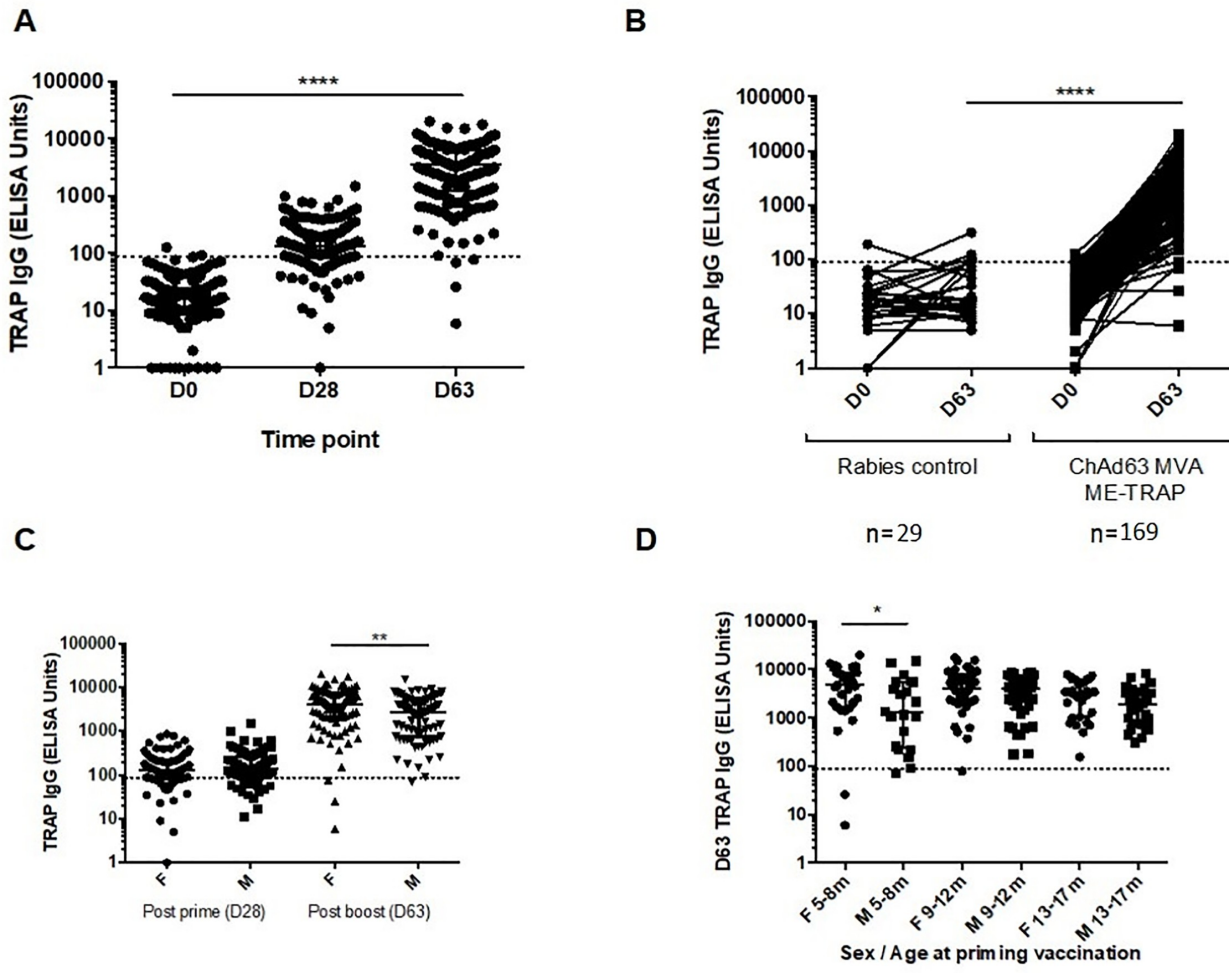
Anti-TRAP IgG titers. **(A)**. Antibody responses to TRAP at baseline, after priming with ChAd63 ME-TRAP (D28) and boosting with MVA ME-TRAP (D63), n = 169, ****p<0.0001, Kruskal-Wallis test. **(B)** Antibody responses at baseline and after second vaccination in the rabies and ME-TRAP vaccinees, (****p<0.0001, Mann-Whitney test). **(C)**Increased antibody titers in female participants after boosting with MVA ME-TRAP, **p<0.004, Mann-Whitney test. **(D)** Increased antibody titers after boosting in the 5–8 months old female children, p = 0.03, Mann-Whitney test.

Antibody responses were measured in 169 children in the ME-TRAP group at pre-vaccination, four weeks after priming with ChAd63 ME-TRAP and one week after boosting with MVA ME-TRAP (Fig 5A). Only two children (1.2%) had positive anti-TRAP IgG titers at baseline. After the priming vaccination, 122 (72%) were seropositive (median titer 136 EUs, 95% CI 118–151) and after boosting, 165 (98%) participants had a positive response with a median titer of 3467 (95% CI 2849–4168 EUs). Responses increased significantly at both post-vaccination time points (p<0.0001, Kruskal-Wallis test with Dunn’s multiple comparison test). Antibody responses were also measured in 29 of the rabies vaccinees at baseline and 9 weeks later (corresponding to the post-MVA time point in the ME-TRAP vaccinees); no significant increase in titer was detected (Fig 4B, p = 0.09, Wilcoxon matched-pairs test) and titers were significantly higher in the ME-TRAP vaccinees than in the rabies group (p<0.0001, Mann-Whitney test). Although not different between sexes after priming vaccination, anti-TRAP IgG titers were significantly higher in female children (p<0.004, Mann-Whitney test, Fig 4C). There was no significant difference in age at the time of first vaccination between male and female children (p = 0.6, Mann-Whitney test), but there was a weak negative correlation between age at vaccination and antibody titer in female children (Spearman’s correlation, r = -0.2, p = 0.2), but not males (Spearman’s correlation, r = -0.08, p = 0.5). When antibody titers were stratified by age and gender, titers were significantly higher in females aged 5–8 months than males of the same age (median titer for females 4763 EUs [95% CI 2117–8461] and 1321 EUs for males [95% CI 257–5486], p = 0.03, Mann-Whitney test, Fig 4D).

### Vaccine efficacy

#### Efficacy against uncomplicated malaria

**Fig 6:**
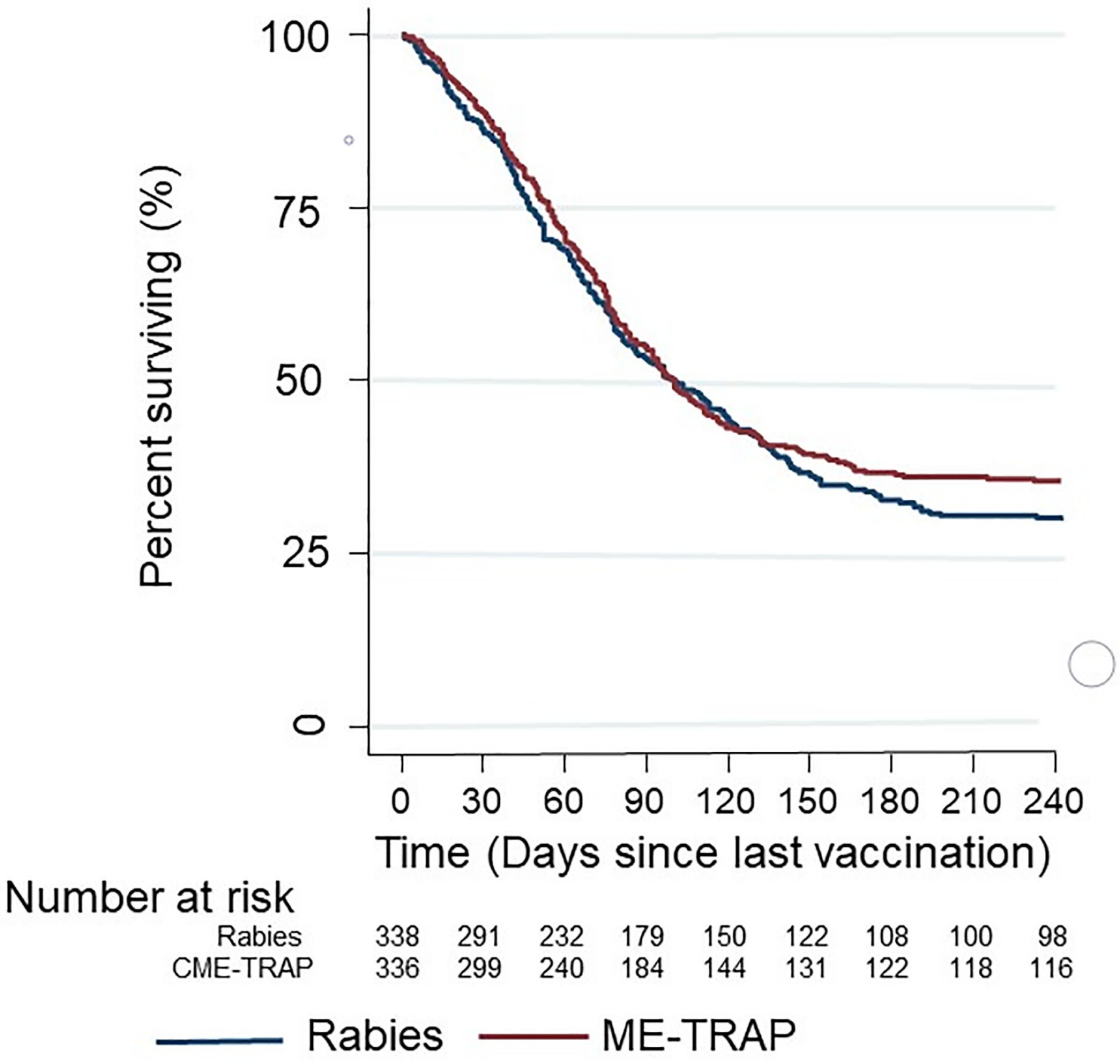
Protective efficacy against clinical malaria of ME-TRAP by Kaplan Meier analysis.

In the per-protocol analysis, 424 children had a first or only clinical malaria episode meeting the primary case definition (212/334 in the ME-TRAP group and 212/336 in the Rabies vaccine group; Fig 6). The proportion of participants with any malaria episode meeting the primary case definition was not significantly different between vaccinees and controls (vaccine efficacy of 13.8%; 95%CI -42.4 to 47.9), and statistically significant efficacy by comparison of proportions was not observed at any timepoint or case definition (S2 Table). There was no significant difference of hazard for malaria episodes between vaccine recipients and control with a Cox hazard ratio (HR) for vaccine efficacy of 3.1% (95%CI -15 to 18.3; p = 0.72) and 4.7% (95%CI -12.4 to 19.1; p = 0.57) respectively in adjusted and unadjusted ATP analyses. The vaccine efficacy estimate did not significantly change for different case definitions based on parasite density cut-offs (S3 Table).

#### Efficacy against severe malaria

**Fig 7:**
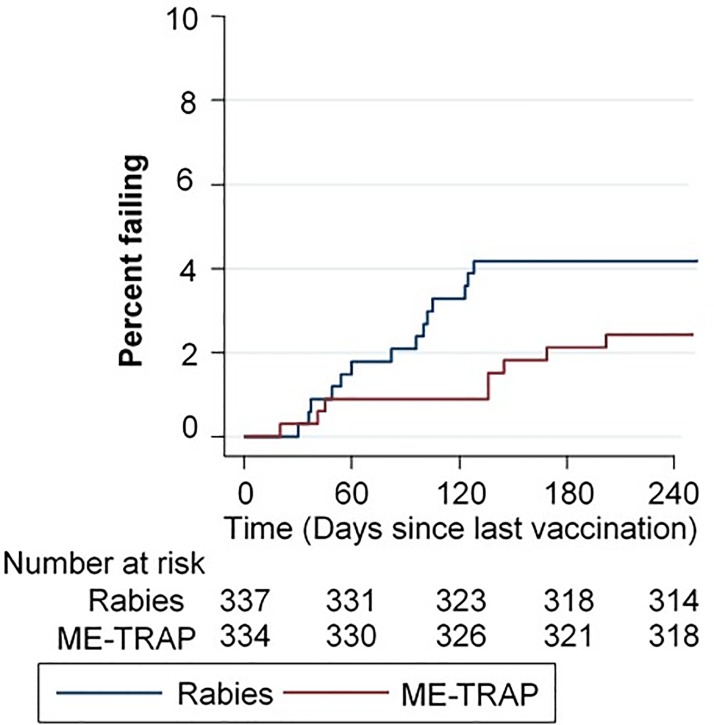
Protective efficacy against severe malaria of ME-TRAP by Kaplan Meier analysis.

Fig 7 shows vaccine efficacy against severe malaria in malaria vaccines recipients and controls. In total 21 vs 17 first episodes of severe malaria occurred in the ITT cohort (Rabies vs ME-TRAP, respectively), whilst there were 20 vs 15 in ATP cohort. In ATP analysis, the vaccine efficacy estimates (Cox regression) against severe malaria was 19.4% (95%CI -58.9 to 59.1; p = 0.53) and -4.7% (95%CI -114.0 to 48.8, p = 0.9) in unadjusted and adjusted cohorts respectively. There were three children who suffered two episodes of severe malaria two from the ME-TRAP arm and one from the rabies arm of the trial. In an exploratory proportions analysis of severe malaria episodes there was some evidence of a reduction in severe malaria episodes at day 360 (VE = 49%, P = 0.14, Fisher’s exact) and at day 120 (VE = 73%, P = 0.03, Fisher’s exact).

## Discussion

This was the first field trial aiming to assess the efficacy, safety and immunogenicity of heterologous prime-boost immunization with the candidate malaria vaccines ChAd63 ME-TRAP and MVA ME-TRAP in children naturally exposed to malaria.

The ChAd63 and MVA ME-TRAP had a similar reactogenicity profile in our malaria exposed infants and children population to that seen in The Gambia who received comparable doses [12,13]. The vaccines demonstrated a good safety profile, inducing only a small number of AEs, all of which were mild in intensity and self-limiting. The variations in creatinine and bilirubin may be chance findings considering that multiple comparisons were conducted at several time points (S3 Table) and would not be of clinical significance in any case.

Although ME-TRAP was shown to be immunogenic, the generated immunological responses were substantially reduced compared to UK, Gambian and Kenyans adult vaccinees who showed peak T cell responses of 1,500–2,300 SFU/ million PBMCs [19,20]. The level of T cell immunogenicity observed in this trial is also substantially below that observed in young Gambian infants immunized with first doses from 1 week of age to four months of age.

Notably, the low T cell responses observed here have been associated with an absence of any sterile protection in several controlled human malaria infection studies of ME-TRAP vaccines in UK trials. Because of the strong correlation of T cell immunogenicity with vaccine efficacy across numerous trials, with significant efficacy when T cell responses exceed 1,500 SFC/ million, it seems very likely that the absence of efficacy against clinical disease in this Burkinabe population results from the very low T cell immunogenicity. Notably, reduced immunogenicity of T cell inducing vaccines, including both viral vectors and whole sporozoite vaccines is generally observed in high transmission malaria areas, such as the trial site in Banfora [21], and in Kenya it was shown that immunogenicity was reduced in children who were recently exposed to malaria [19]. One possible solution would be to administer vectored vaccines to young infants before they are susceptible to malaria-induced immunosuppression. The recent observation of potent T cell immunogenicity in young Gambian infants supports the potential of this approach [14].

Antibody responses to TRAP were significantly boosted by both ChAd63 and MVA ME-TRAP and were higher than those induced in either UK or Gambian adults as we have previously demonstrated in a lead-in safety study [13]. In the absence of significant efficacy, the contribution of antibody responses to TRAP is impossible to determine, however we have previously demonstrated a potential modest effect of anti-TRAP antibodies in reducing parasite density during liver-stage infection [22]. This additional component of immunity could therefore contribute to vaccine efficacy in regimes administered to young children.

Despite the absence of statistically significant efficacy against clinical malaria, an intriguing finding was the observation of a marginally significant reduction in cases of severe malaria amongst malaria vaccinees at day 120 of follow-up in a secondary analysis of severe malaria as an endpoint. This could be a chance finding and the trial was not powered to measure efficacy against severe malaria. Alternatively, if a true finding, this could indicate that T cell inducing vaccines against liver-stage malaria will have greater efficacy against severe malaria than against clinical malaria. It is worth noting that there have been previous considerations of the potentially greater efficacy of liver-stage vaccines against severe malaria based on finding that there is a stronger reported HLA class I association with severe malaria than clinical malaria [23–25].

Although in theory, very high levels of pre-existing anti-vector immunity could limit the immunogenicity of adenoviral vectored vaccines in exposed populations, only low prevalence of neutralizing antibodies to ChAd63 are observed in populations living in the study area both in adults and children [17], and so this seems unlikely to explain reduced immunogenicity. Furthermore, the modest levels of neutralizing antibodies to ChAd63 observed also in UK adults did not impact vaccine immunogenicity and efficacy in UK adult vaccinees [8].

Overall, these study findings further support the safety of vectored vaccines when used in African infants and children but highlight the need to find either immunization time points, such as early infancy, where the high T cell immunogenicity required for vaccine efficacy can be induced, or, alternatively, immunization regimens that better target T cells to the liver [26]. This study suggests that vaccination needs to take place prior to the onset of what appears to be substantial immunosuppression of vaccine-induced T cell immunogenicity in parallel with increased malaria exposure.

## Supporting information

S1 CONSORT 2010 Checklist (DOC)

S1 Protocol Trial protocol. (PDF)

S1 Table Laboratory safety: Median values. (DOCX)

S2 Table Vaccine efficacy by tests of proportions. (DOCX)

S3 Table Vaccine efficacy by Cox regression. (DOCX)
