# Supplementary material for: First field efficacy trial of the ChAd63 MVA ME-TRAP vectored malaria vaccine candidate in 5-17 months old infants and children
Source: PLoS One. 2018 Dec 12;13(12):e0208328. doi: 10.1371/journal.pone.0208328 (PMC6291132; doi:10.1371/journal.pone.0208328)
Supplement: S1 Table — (DOCX) [file pone.0208328.s003.docx]

### **S1 Table:** Laboratory safety: median values

|  |  | **ME-TRAP** | | | | **Rabies** | | | |  |
| --- | --- | --- | --- | --- | --- | --- | --- | --- | --- | --- |
| **Parameter** | **Visit** | **5^th^ Centile** | **50^th^ Centile** | **95^th^ Centile** | **N** | **5^th^ Centile** | **50^th^ Centile** | **95^th^ Centile** | **N** | **P** |
| ALT | Visit 06 | 6.7 | 12 | 28 | 312 | 6 | 13 | 25.3 | 313 | .27 |
| Bilirubin | Visit 06 | 6 | 12.4 | 27.5 | 312 | 5.9 | 11.6 | 29 | 313 | .28 |
| Creatinine | Visit 06 | 20.4 | 29.8 | 36.8 | 312 | 21 | 29.5 | 38.4 | 313 | .6 |
| Haemoglobin | Visit 06 | 7.4 | 9.3 | 10.9 | 312 | 7.7 | 9.4 | 10.9 | 307 | .049 |
| Neutrophils | Visit 06 | 1.2 | 2.5 | 5.6 | 312 | 1.3 | 2.6 | 5.1 | 307 | .32 |
| Platelets | Visit 06 | 100.4 | 396 | 662 | 312 | 102.4 | 395 | 641.4 | 307 | .84 |
| White blood cells | Visit 06 | 6.8 | 10.8 | 18.2 | 312 | 7.2 | 10.9 | 17.8 | 307 | .78 |
| ALT | Visit 11 | 7 | 13 | 33.8 | 323 | 7 | 13 | 30.5 | 308 | .49 |
| Bilirubin | Visit 11 | 6.1 | 13.5 | 32.1 | 323 | 6.2 | 12.7 | 31.4 | 308 | .36 |
| Creatinine | Visit 11 | 21 | 31.2 | 41.6 | 323 | 20 | 30.2 | 40 | 309 | .017 |
| Haemoglobin | Visit 11 | 7.2 | 9.4 | 11 | 313 | 7.6 | 9.5 | 11.1 | 308 | .12 |
| Neutrophils | Visit 11 | 1.1 | 2.4 | 5.7 | 313 | 1 | 2.5 | 5.6 | 308 | .4 |
| Platelets | Visit 11 | 84.7 | 392 | 658 | 313 | 90.9 | 380 | 651 | 308 | .4 |
| White blood cells | Visit 11 | 6.7 | 10.8 | 18.2 | 313 | 6.5 | 10.3 | 17.3 | 308 | .08 |
| ALT | Visit 17 | 6 | 12 | 29.8 | 324 | 6 | 11 | 31 | 327 | .33 |
| Bilirubin | Visit 17 | 2.9 | 9 | 23.3 | 322 | 2.9 | 8 | 25.2 | 327 | .017 |
| Creatinine | Visit 17 | 22.7 | 34.5 | 47.7 | 324 | 19.7 | 32.9 | 48.6 | 327 | .007 |
| Haemoglobin | Visit 17 | 6.8 | 9 | 10.7 | 324 | 6.7 | 9 | 10.8 | 322 | .53 |
| Neutrophils | Visit 17 | 1.1 | 2.4 | 4.8 | 324 | 1.1 | 2.3 | 5 | 322 | .12 |
| Platelets | Visit 17 | 104.3 | 337.5 | 572.5 | 324 | 121 | 327.5 | 569.5 | 322 | .16 |
| White blood cells | Visit 17 | 6 | 9.3 | 15.6 | 324 | 5.7 | 8.8 | 14.6 | 322 | .06 |
