# Supplementary material for: First field efficacy trial of the ChAd63 MVA ME-TRAP vectored malaria vaccine candidate in 5-17 months old infants and children
Source: PLoS One. 2018 Dec 12;13(12):e0208328. doi: 10.1371/journal.pone.0208328 (PMC6291132; doi:10.1371/journal.pone.0208328)
Supplement: S2 Table — (DOCX) [file pone.0208328.s004.docx]

**S2 Table:** Vaccine efficacy by tests of Proportions

| Cohort | Endpoint | Day _(t)_ | Rabies | | | ME-TRAP | | | Efficacy | | |
| --- | --- | --- | --- | --- | --- | --- | --- | --- | --- | --- | --- |
|  |  |  | S*_t_* | lb | ub | S_t_ | lb | ub | Estimate (%) | lb | ub |
| ATP | >5000 & >37.5 (Primary) | 90 | 53.1 | 47.6 | 58.3 | 54.4 | 48.9 | 59.6 | 2.4 | -34.8 | 29.3 |
| ATP | >0 & >37.5/Hx fever | 90 | 48.4 | 42.9 | 53.6 | 51.4 | 46 | 56.6 | 6 | -33.3 | 33.7 |
| ATP | >500 & >37.5/Hx fever | 90 | 46 | 40.6 | 51.2 | 49.3 | 43.9 | 54.6 | 6.8 | -34.3 | 35.3 |
| ATP | >20,000 & >37.5/Hx fever | 90 | 62.6 | 57.2 | 67.5 | 62.8 | 57.3 | 67.7 | .2 | -30.5 | 23.7 |
| ITT | >5000 & >37.5 (Primary) | 90 | 52.2 | 46.7 | 57.4 | 54.1 | 48.7 | 59.3 | 3.6 | -33.6 | 30.4 |
| ITT | >0 & >37.5/Hx fever | 90 | 48.9 | 43.5 | 54.1 | 50 | 44.5 | 55.2 | 2.1 | -39 | 31.1 |
| ITT | >500 & >37.5/Hx fever | 90 | 45.4 | 40 | 50.6 | 47.6 | 42.2 | 52.8 | 4.7 | -38.4 | 34.3 |
| ITT | >20,000 & >37.5/Hx fever | 90 | 61.9 | 56.5 | 66.9 | 62.4 | 57 | 67.3 | .8 | -30.1 | 24.3 |
| ATP | >5000 & >37.5 (Primary) | 180 | 32.8 | 27.8 | 37.8 | 36.7 | 31.6 | 41.9 | 10.7 | -44.2 | 44.7 |
| ATP | >0 & >37.5/Hx fever | 180 | 28.3 | 23.6 | 33.2 | 29.8 | 25 | 34.8 | 5 | -63.9 | 44.9 |
| ATP | >500 & >37.5/Hx fever | 180 | 26.2 | 21.7 | 31 | 28.9 | 24.2 | 33.9 | 9.3 | -59.8 | 48.5 |
| ATP | >20,000 & >37.5/Hx fever | 180 | 45.3 | 39.9 | 50.5 | 46 | 40.6 | 51.2 | 1.5 | -44.1 | 32.7 |
| ITT | >5000 & >37.5 (Primary) | 180 | 32.8 | 27.8 | 37.8 | 34.4 | 29.4 | 39.5 | 4.7 | -55.4 | 41.6 |
| ITT | >0 & >37.5/Hx fever | 180 | 27.4 | 22.8 | 32.3 | 27 | 22.3 | 31.8 | -1.7 | -79.9 | 42.5 |
| ITT | >500 & >37.5/Hx fever | 180 | 25.7 | 21.1 | 30.4 | 26.1 | 21.5 | 30.9 | 1.5 | -77.6 | 45.4 |
| ITT | >20,000 & >37.5/Hx fever | 180 | 44.6 | 39.3 | 49.9 | 43.6 | 38.3 | 48.8 | -2.4 | -51.4 | 30.8 |
| ATP | >5000 & >37.5 (Primary) | 270 | 30.3 | 25.5 | 35.3 | 35.2 | 30.1 | 40.3 | 13.8 | -42.4 | 47.9 |
| ATP | >0 & >37.5/Hx fever | 270 | 25.9 | 21.3 | 30.6 | 27.4 | 22.7 | 32.2 | 5.5 | -68.9 | 47.1 |
| ATP | >500 & >37.5/Hx fever | 270 | 24.1 | 19.6 | 28.8 | 27.4 | 22.7 | 32.3 | 12.1 | -59.4 | 51.5 |
| ATP | >20,000 & >37.5/Hx fever | 270 | 42.5 | 37.2 | 47.8 | 43.2 | 37.9 | 48.5 | 1.6 | -47.2 | 34.2 |
| ITT | >5000 & >37.5 (Primary) | 270 | 28.8 | 24.1 | 33.8 | 32.6 | 27.6 | 37.6 | 11.5 | -49.5 | 47.7 |
| ITT | >0 & >37.5/Hx fever | 270 | 24.4 | 19.9 | 29.1 | 25.7 | 21.2 | 30.5 | 5.3 | -73.3 | 48.2 |
| ITT | >500 & >37.5/Hx fever | 270 | 22.6 | 18.3 | 27.2 | 25.1 | 20.6 | 29.9 | 10.1 | -67.8 | 51.8 |
| ITT | >20,000 & >37.5/Hx fever | 270 | 41.6 | 36.3 | 46.8 | 41.2 | 35.9 | 46.4 | -1 | -52.9 | 33.2 |

S*_t_* Is the proportion surviving without the endpoint indicated to time *t***.**  lb & ub are the 95% lower and upper bounds of the estimated S_t_ and efficacies.

ATP: According to Protocol; ITT: Intent to Treat; Hx: History of fever
