# Supplementary material for: First field efficacy trial of the ChAd63 MVA ME-TRAP vectored malaria vaccine candidate in 5-17 months old infants and children
Source: PLoS One. 2018 Dec 12;13(12):e0208328. doi: 10.1371/journal.pone.0208328 (PMC6291132; doi:10.1371/journal.pone.0208328)
Supplement: S3 Table — (DOCX) [file pone.0208328.s005.docx]

**S3 Table:** Vaccine efficacy by Cox regression

| **Cohort** | **Endpoints** | **Adjusting** | **Efficacy (%)** | **LB** | **UB** | **p** |
| --- | --- | --- | --- | --- | --- | --- |
| ATP | >5000 & >37.5 (Primary) | adjusted | 3.1 | -15.0 | 18.3 | .72 |
| ATP | >5000 & >37.5 (Primary) | unadjusted | 4.7 | -12.4 | 19.1 | .57 |
| ATP | >0 & >37.5/Hx fever | adjusted | -.2 | -18.4 | 15.3 | .98 |
| ATP | >0 & >37.5/Hx fever | unadjusted | -.2 | -17.7 | 14.6 | .98 |
| ATP | >500 & >37.5/Hx fever | adjusted | 4.8 | -12.6 | 19.5 | .56 |
| ATP | >500 & >37.5/Hx fever | unadjusted | 5.7 | -10.7 | 19.7 | .47 |
| ATP | >20,000 & >37.5/Hx fever | adjusted | .1 | -19.4 | 16.3 | .99 |
| ATP | >20,000 & >37.5/Hx fever | unadjusted | -.5 | -19.2 | 15.3 | .96 |
| ITT | >5000 & >37.5 (Primary) | adjusted | 1.8 | -16.4 | 17.2 | .83 |
| ITT | >5000 & >37.5 (Primary) | unadjusted | 3.5 | -13.7 | 18.1 | .67 |
| ITT | >0 & >37.5/Hx fever | adjusted | -3.6 | -22.3 | 12.3 | .68 |
| ITT | >0 & >37.5/Hx fever | unadjusted | -3 | -20.8 | 12.2 | .72 |
| ITT | >500 & >37.5/Hx fever | adjusted | 2.5 | -15.2 | 17.6 | .76 |
| ITT | >500 & >37.5/Hx fever | unadjusted | 4.1 | -12.5 | 18.3 | .61 |
| ITT | >20,000 & >37.5/Hx fever | adjusted | -1.9 | -21.7 | 14.6 | .83 |
| ITT | >20,000 & >37.5/Hx fever | unadjusted | -2.2 | -21.1 | 13.8 | .8 |

lb & ub are the 95% lower and upper bounds of the estimated efficacy. ATP: According to Protocol; ITT: Intent to Treat; Hx: History of fever
